# Unveiling the Role of Gut Microbiota and Metabolites in Autoimmune Thyroid Diseases: Emerging Perspectives

**DOI:** 10.3390/ijms252010918

**Published:** 2024-10-10

**Authors:** Kai Yan, Xin Sun, Chenxi Fan, Xin Wang, Hongsong Yu

**Affiliations:** 1 Department of Immunology, Special Key Laboratory of Ocular Diseases of Guizhou Province, Zunyi Medical University, Zunyi 563000, China; yankai0909@163.com (K.Y.); fcx981118@163.com (C.F.); 2 School of Basic Medical Sciences, Special Key Laboratory of Gene Detection and Therapy of Guizhou Province, Zunyi Medical University, Zunyi 563000, China; sunxinzmu@163.com (X.S.); wangxin@zmu.edu.cn (X.W.)

**Keywords:** thyroid, gut microbiota, AITDs, SCFAs, trace elements

## Abstract

Autoimmune thyroid diseases (AITDs) are among the most prevalent organ-specific autoimmune disorders, with thyroid hormones playing a pivotal role in the gastrointestinal system’s structure and function. Emerging evidence suggests a link between AITDs and the gut microbiome, which is a diverse community of organisms that are essential for digestion, absorption, intestinal homeostasis, and immune defense. Recent studies using 16S rRNA and metagenomic sequencing of fecal samples from AITD patients have revealed a significant correlation between a gut microbiota imbalance and the severity of AITDs. Progress in animal models of autoimmune diseases has shown that intervention in the gut microbiota can significantly alter the disease severity. The gut microbiota influences T cell subgroup differentiation and modulates the pathological immune response to AITDs through mechanisms involving short-chain fatty acids (SCFAs), lipopolysaccharides (LPSs), and mucosal immunity. Conversely, thyroid hormones also influence gut function and microbiota composition. Thus, there is a bidirectional relationship between the thyroid and the gut ecosystem. This review explores the pathogenic mechanisms of the gut microbiota and its metabolites in AITDs, characterizes the gut microbiota in Graves’ disease (GD) and Hashimoto’s thyroiditis (HT), and examines the interactions between the gut microbiota, thyroid hormones, T cell differentiation, and trace elements. The review aims to enhance understanding of the gut microbiota–thyroid axis and proposes novel approaches to mitigate AITD severity through gut microbiota modulation.

## 1. Introduction

**Figure 1:**
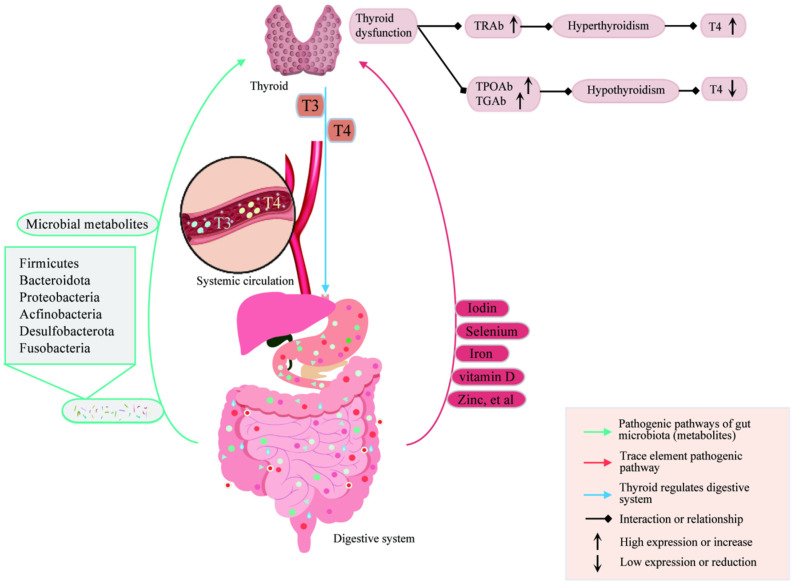
The intestinal microbiota interact with the thyroid. The combined effects of the intestinal microbiota and their metabolites, trace element absorption and utilization, thyroid function, and hormone levels lead to the occurrence of AITDs.

Autoimmune thyroid diseases (AITDs) are significant autoimmune disorders affecting thyroid function, which are diagnosed by the presence of autoantibodies against thyroid antigens, such as thyroglobulin antibodies (TGAbs) and thyroid microsomal antibodies (TMABs) [1]. The most common types of AITDs are Graves’ disease (GD) and Hashimoto’s thyroiditis (HT). GD, also known as toxic diffuse goiter, is caused by autoantibodies that stimulate the thyroid-stimulating hormone receptor (TSHR) [2], leading to symptoms such as thyroid enlargement, hypermetabolism, anxiety, irritability, and in severe cases, thyroid storm [3]. HT is the primary cause of hypothyroidism and is characterized by positive thyroid peroxidase (TPO) antibodies (TPOAbs) and TGAbs [4,5]. T cell differentiation significantly influences the adaptive immune response, and T lymphocyte-mediated destruction of thyroid follicles and immune dysfunction is critical in the development of AITDs [6], as Th1 cells produce pro-inflammatory cytokines, while Th2 cells release anti-inflammatory cytokines, moderating Th1 activity in AITDs [7]. Additionally, AITD patients show an increased proportion of pro-inflammatory Th17 cells and a significant decrease in regulatory T (Treg) cells in their peripheral blood mononuclear cells (PBMCs) [8]. This imbalance results in a substantial shift in the Th17/Treg ratio, worsening inflammation severity in AITDs. The gut, housing the most extensive microbial community within the human digestive system, is vital for nutrient and drug absorption (Figure 1) [9]. The immune defenses in the gut, mediated by the microbiota, acts as a critical biological barrier, maintain stability in both the systemic and enterohepatic circulation [10]. Different T cell subsets within the gut-associated lymphoid tissue, such as Th1, Th2, Th17, and Treg cells, play essential roles in autoimmune diseases, as the balance of Th1/Th2 and Th17/Treg cells is influential in the progression of autoimmune diseases like rheumatoid arthritis, systemic lupus erythematosus (SLE), and Crohn’s disease. The study of gut microbiota is mainly focused on 16S and genomic sequencing, primarily at the genus and species level. The human gut microbiota is predominantly composed of the phyla *Firmicutes* and *Bacteroidetes* [11]. Significant variations in the Firmicutes/Bacteroidetes (F/B) ratio are observed in autoimmune conditions such as inflammatory bowel disease, autoimmune hepatitis, and systemic lupus erythematosus, as compared to healthy controls [12]. Patients with AITDs also show similar F/B ratio changes.

There is an interaction between the thyroid and the gut, with AITD patients often exhibiting symptoms of celiac disease (CD), particularly in cases of HT and GD. The thyroid is essential for human development, and its dysfunction can lead to developmental delays [13,14]. Thyroid hormones impact the structure and function of the gastrointestinal tract by influencing the development and differentiation of intestinal epithelial cells [15]. Similarly, the gut microbiota plays an important role in thyroid health. *Collinsella* and *Roseburia* have been identified as key gut microbial markers in patients with AITDs, while *Butyricimonas* has been identified in patients with GD and HT. Despite these findings, there is limited evidence regarding the mechanism by which the gut microbiome is correlated with AITDs, GD, and HT. The gut microbiota, along with other pathogenic factors, influences the progression of these diseases, but it is not directly involved in their causal mechanisms. The impact of the gut microbiota on AITDs can be evaluated from various perspectives, including gut immune barrier disruption, T cell differentiation imbalance, and disturbances in the absorption and utilization of trace elements. This review aims to provide new insights into the treatment of AITDs and the development of a gut–thyroid axis framework.

## 2. Clinical and Immune Characteristics of AITDs

Abnormal clinical test indicators are significant markers of disease progression in AITD patients. TPOAbs are a standard marker for diagnosing thyroid disease, while TMABs are essential for distinguishing AITDs and act as a specific marker for chronic lymphocytic thyroiditis. Thyroid receptor antibodies (TRAbs) target the TSHR directly on thyroid cell membranes. Free Triiodothyronine (FT3) and Free Thyroxine (FT4) are sensitive indicators for diagnosing hypothyroidism. Sequencing studies have identified substantial correlations between the gut microbiota abundance and thyroid hormone markers, including TRAbs, TGAbs, TPOAbs, FT3, and FT4 [16,17,18]. AITDs are characterized by an imbalance of immune cells. Th17 cells promote inflammation by releasing interleukin (IL)-17, while Treg cells, identified by the transcription factor FoxP3, maintain immunological stability by producing inhibitory cytokines such as transforming growth factor-beta (TGF-β), IL-10, and IL-35 [19]. These cells also help generate and differentiate anti-inflammatory cytokines through T-cell receptor (TCR) signaling [20]. In GD patients, Treg cells and FoxP3 mRNA expression levels are significantly reduced [21], resulting in a noticeable decrease in the Treg cell count. Similarly, individuals with HT exhibit thyroid infiltration by T and B cells, particularly CD4^+^ Th1 cells, indicating the crucial role of T cell differentiation imbalances in the development of AITDs.

GD is a primary autoimmune thyroid disorder characterized by the production of serum thyroid-stimulating antibodies (TSAbs) in patients. Common symptoms include thyroid enlargement, hyperthyroidism, pretibial myxedema, heightened metabolic rates, and ocular issues such as exophthalmos. Systemic symptoms like palpitations, irritability, increased appetite, and weight loss are also frequently observed [22]. Diagnosis is primarily based on clinical features, thyroid-stimulating hormone (TSH) levels, free thyroid hormone levels, and TRAb levels. The immunological characteristics of GD mainly manifest as an abnormal distribution of T cell subgroups, which produce autoantibodies against the thyroid tissue, including TRAbs, TPOAbs, and TGAbs. Patients with GD have elevated levels of TRAbs, which exhibit a high affinity for the TSHR, leading to the unregulated high secretion of thyroid hormones [23]. When TRAbs interact with the TSHR on thyroid cell membranes, they excessively activate the TSHR, causing diffuse thyroid goiter and excessive thyroid hormone production. In autoimmune conditions, an imbalance between Th17 and Treg cells can cause inflammatory disorders. Studies have shown that in patients with GD, there is increased differentiation of Th17 cells, which promotes the secretion of immune cytokines such as IL-17 [24]. Conversely, there is a decrease in Treg cell expression, leading to lower levels of IL-10 [25]. The levels of TSAbs are also positively associated with IL-17, IL-22, and the number of Th17 cells [26]. These changes in the Th17/Treg ratio indicate different inflammatory states in GD and can serve as an important immune checkpoint.

HT, also known as chronic lymphocytic thyroiditis, is the primary cause of hypothyroidism. It is characterized by the immune system attacking the thyroid gland, causing a thyroid immune imbalance and follicular cell dysfunction. The onset of HT involves a complex interplay of genetic susceptibility, environmental factors, and autoimmune mechanisms, with thyroid dysfunction primarily indicated by positive TPOAbs and TGAbs [27]. Hypothyroidism is identified by elevated TSH levels and reduced serum-free thyroxine (T4) levels [28]. Patients with subclinical hypothyroidism (SCH) exhibit elevated TSH levels while maintaining normal T4 counts. Mild subclinical hypothyroidism can lead to disorders in lipid metabolism and increase the risk of cardiovascular and cerebrovascular diseases. Patients with severe SCH typically undergo L-thyroxine replacement therapy. During AITD pathogenesis, lymphocytes infiltrate the thyroid tissue, and activated autoreactive CD4^+^ T cells recruit CD8^+^ T cells and B cells to attack the thyroid cells with autoantibodies [29]. The elevated levels of Th1 and Th17 cells in HT patients lead to thyroid inflammatory immune cell infiltration [30]. Excessive iodine intake can heighten the immune response to thyroglobulin, leading to HT, while a lack of selenium may reduce protection against TPOAbs production, disrupting the TPOAbs balance. In addition to iodine, liver processes such as sulfation and glucuronidation may esterify phenolic hydroxyl groups with sulfate or glucuronic acid, increasing the water solubility of iodinated thyroglobulin and playing a role in the metabolism of thyroid hormones [31]. These processes, along with thyrotropin-releasing hormone, TSH, gut microbiota, and other molecules, jointly regulate thyroid function [32].

## 3. Characteristics of Gut Microbiota in AITDs

Microbiota dysbiosis significantly impacts the immune system and the regulation of inflammation. In AITDs, the disrupted gut microbiota alters metabolite circulation [33], influencing the absorption and utilization of thyroid micronutrients [34]. Studies have identified specific bacterial signatures, such as *Bacteroides*, *Prevotella*, and *Alistipes*, that distinguish patients from healthy individuals [35]. The alpha diversity of the gut microbiota, often measured using indices such as Chao1, Shannon-wiener, Simpson, Good’s Coverage, and Phylogenetic Diversity [36,37], is typically lower in patients with GD compared to healthy individuals [38]. Beta diversity analyses, including Principal Component Analysis and Principal Coordinates Analysis, also show distinct differences between GD patients and healthy populations, despite some overlap [39]. Geographical variations in the gut microbiota have been observed across European, American, and Asian populations, likely influenced by diet and lifestyle. For example, differences in the Shannon-wiener index of alpha diversity have been noted between German and United Kingdom populations, while Belgium shows a higher F/B ratio at the phylum level compared to the United Kingdom, suggesting a predominance of *Firmicutes* in the Belgian population [40]. Two-sample Mendelian randomization studies have identified a significant link between GD and specific gut microbial communities, including *Deltaproteobacteria*, *Mollicutes*, *Ruminococcus torques group*, *Oxalobacter*, and *Ruminococcaceae* UCG 011, suggesting a close relationship with the onset of hyperthyroidism [41]. Comparative analyses between GD and HT show no significant difference at the phylum level, however, notable genus-level differences exist. *Prevotella* 9 is more abundant in GD, whereas *Roseburia* is more prevalent in HT [42]. Subsequent research has further investigated the differences in the abundance of gut microbiota in GD at both the phylum and genus levels [43]. Research results show that the proportion of *Firmicutes* is higher than that of *Bacteroidota* and *Actinobacteria* [16,39]. Additionally, the changes in abundance at the genus level are mainly between *Firmicutes* and *Bacteroidota* [44,45,46]. Detailed information is summarized in Table 1.

The gut microbiota interacts with epithelial and mucosal immune cells through pattern recognition receptors (PRRs), which can activate pro-inflammatory or anti-inflammatory factors and influence the functionality of the immune system [37]. The small intestine epithelium, as the largest mucosal immune barrier, is particularly affected when mucosal inflammation and leaky gut syndrome occur. This condition allows for immunogenic inflammatory factors to pass through the mucosal barrier, potentially leading to systemic inflammation. Increased intestinal permeability may contribute to systemic inflammation and possibly influence the development of AITDs, although further research is needed to establish a causal relationship [33]. Treatment for HT primarily involves lifelong oral administration of levothyroxine to maintain normal hormone levels. The gut microbiota can influence thyroid hormone levels through the absorption and metabolism of levothyroxine [27,48]. In HT patients, while the Firmicutes phylum remains the most abundant at the phylum level [34], its overall diversity and abundance are significantly reduced compared to healthy controls [49,50]. Similar characteristics have been observed in GD patients. Differences also exist in the abundance of horizontal gene groups within the Firmicutes and Bacteroidetes phyla [51]. Beneficial bacteria such as lactic acid bacteria and *Bifidobacteria* decrease [52], while harmful microbial populations like *Bacteroides fragilis* increase significantly [53,54]. The gut microbiota and their byproducts can greatly affect HT pathogenesis by influencing intestinal immune defense and the absorption of medications and trace elements (Table 2).

## 4. Progress of Gut Microbiota in Animal Models of Autoimmune Diseases

Animal models are crucial for identifying and validating factors related to disease development. In mouse models of inflammatory bowel disease, butyrate promotes IL-10 secretion in the intestinal mucosa and inhibits IL-17 and IL-22 production [55]. In colitis mouse models, interventions with bacterial groups such as *Faecalibacterium prausnitzii* and *Clostridium butyricum* increased levels of SCFAs as well as Treg cells, and decreased Th17 cells [53]. Studies have shown that *Prevotella* differs significantly in patients with AITDs and alleviates the severity of mouse arthritis through butyrate levels [54]. In experimental autoimmune encephalomyelitis mouse studies, long-chain fatty acids increase pro-inflammatory Th1 and Th17 cell differentiation, while SCFAs promote the differentiation of Treg cells by inhibiting the JNK1 and p38 signaling pathways [56]. In SLE mice, butyrate and propionate in drinking water improve lupus-induced skin and kidney lesions by inhibiting histone deacetylases (HDACs) [57]. Additionally, feeding mice with acetic acid-modified high-amylose maize-resistant starch reduces the frequency of autoreactive T cells in lymphoid tissues and diabetes-related cytokines in serum [58].

Creating a GD mouse model involves immunizing female BALB/c mice with an adenovirus carrying the human TSHR A-subunit gene (Ad-TSHR289) [59]. TSHR antibodies can cause hyperthyroidism [60], successfully producing a GD mouse model. TSHR, a G protein-coupled receptors (GPCRs) on thyroid follicular cells, is the autoantigen targeted by antibodies in GD [61]. This immunization leads to significantly higher T4 and thyroid-stimulating TRAb levels [62]. Histologically, GD models exhibit enlarged thyroids, increased thyroid cell size, epithelial hyperplasia, and notable follicular changes such as vacuolization [63], corresponding to the pathological changes seen in GD patients. Flow cytometry revealed a reduced proportion of CD4^+^CD25^+^FoxP3^+^ Treg cells in the Ad-TSHR289 group compared to the Ad-Control group [64]. This immunization may disrupt the gut microbiota balance by affecting both thyroid function and hormone levels. Research on GD mouse models shows significantly decreased alpha diversity and *Firmicutes* abundance compared to control groups, with reduced *Akkermansia* and the *Lachnospiraceae NK4A136 group* at the genus level, and increased *Faecalibacterium*, *Ruminococcaceae*, *Lactobacillus*, and *Odoribacter* [65]. Beta diversity analyses further emphasize the differences in microbial populations. GD mice receiving gut microbiota transplants from GD patients show higher model establishment success rates compared to those receiving transplants from healthy individuals. Post-transplant, TSHR immunization increases the total T4, TRAbs, and IL-17A levels, while reducing the IL-10 serum concentrations [66]. These findings highlight the crucial role of the gut microbiota in GD progression and suggest its potential as a critical target for modulating disease severity and immune responses. Recent research shows that selenium supplementation in a GD mouse model increases gut microbiota diversity, promotes beneficial microbial populations, and reduces pathogenic ones [67]. This underscores the importance of the gut microbiota in absorbing and utilizing essential trace elements for thyroid health. Additionally, BALB/cJ mice injected with TSHR-Abs on a vitamin D-deficient diet showed higher susceptibility to sustained hyperthyroidism compared to those on a standard diet. [68]. NOD1 protein, a member of the NOD-like receptor (NLR) family, triggers chemokine production and acute inflammatory cell recruitment in high-fat diet mice through NOD1 activation in intestinal epithelial cells, leading to increased free thyroid hormone T4 levels [69]. In the study of gut microbiota in GD mouse models, the sequencing results of the gut microbiota demonstrated characteristics consistent with those of humans. These findings highlight the importance of further investigating the effects of the gut microbiota on the immune system and thyroid function in GD animal models, particularly through dietary interventions such as probiotics and prebiotics.

## 5. Involvement of Gut Microbiota and SCFAs in AITDs through Th17/Treg Balance

### 5.1. Gut Microbiota Is Significantly Associated with Immune Regulation

The intestinal barrier is composed mainly of the intestinal epithelium, a mucus layer, and gut-associated lymphoid tissues. Imbalances in the gut microbiota and their metabolites can disrupt gut mucosa function and immune cell expression in mesenteric lymph nodes. An increase in pathogenic gut bacteria can enhance the release of inflammatory factors, impair the gut mucosa’s defensive function, affect immune tolerance, and promote systemic inflammation [70]. The gut microbiota is crucial for immune function. Immunoglobulins like IgA and IgG are secreted into the intestinal mucosa, interacting with the gut microbiota to help maintain immune homeostasis. This interaction leads to the generation of inflammatory cytokines, such as tumor necrosis factor-alpha (TNF-α), IL-1, and IL-6, which are crucial for immunity [71]. For instance, in humans, a *Yersinia enterocolitica* infection can trigger the generation of lipoprotein antibodies that inhibit the functionality of Treg cells. In rheumatoid arthritis, *Collinsella* has been shown to be associated with elevated production of the pro-inflammatory cytokine IL-17A [72]. In addition, *Bacteroides* are positively correlated with CD4^+^ CD25^+^ Treg cells, Th2 cells, and serum IL-10 levels, but negatively correlated with Th1 cells, the Th1/Th2 ratio, and serum TNF-α and interferon-gamma (IFN-γ) levels [73]. Conversely, *Prevotella* is positively correlated with Th1 cells, the Th1/Th2 ratio, and serum TNF-α levels. *Yersinia enterocolitica*, segmented filamentous bacteria, and *Eubacterium rectale* positively correlate with the Th17/Treg ratio, while *Bifidobacteria*, *Lactobacilli*, and *Prevotella* exhibit a negative correlation. *Eubacterium* also significantly negatively correlates with IL-17 levels [35]. Changes in intestinal mucosal permeability can upregulate IL-17 levels via *Collinsella* [74]. *P. prausnitzii* has been shown to encourage monocytes to increase the secretion of the anti-inflammatory cytokine IL-10 via leukocytes [75]. Therefore, the gut microbiota play an important role in the occurrence and development of AITDs, possibly by regulating intestinal homeostasis and T cell differentiation [76].

### 5.2. Gut Microbiota Regulates the Synthesis and Metabolism of SCFAs

The gut microbiota facilitates the production of SCFAs, such as acetate, propionate, and butyrate, through the fermentation and conversion of dietary carbohydrates via microbial glycolysis. Various microbes contribute uniquely to SCFA production. For instance, *Intestinimonas* produces acetate, propionate, and butyrate; *Phascolarctobacterium* synthesizes acetate and propionate [77]; *B. breve* CECT7263 and *Coprococcus* sp. are sources of acetate [78]; *Roseburia* produces acetate and butyrate; and *Bacteroides* generates propionate and acetate [47,79]. *Bacteroides fragilis* YCH46 not only produces propionate but also increases Treg cell numbers while decreasing Th17 cell populations [66]. Other microbes, such as *Prevotella*, *Ruminococcus*, and *Veionella,* have been identified as propionate producers [80]. Conversely, groups including *Butyrivibrio*, *Coprococcus*, *Eubacterium eligens*, *Blautia*, *Faecalibacterium*, *Clostridium cluster XVIa*, and *F. prausnitzii* are known for butyrate production [81,82]. *Anaerostipes*, *Coprococcus*, *Subdoligranulum*, and *Pseudobutyrivibrio* also produce butyrate [83,84]. Acetic acid, propionic acid, and butyric acid account for more than 90% of SCFAs. Disturbances in intestinal homeostasis reduce diversity, decrease beneficial bacteria, and increase pathogenic bacteria, leading to lower levels of SCFAs in the body.

### 5.3. Involvement of SCFAs in AITDs through Regulation of T Cell Differentiation

**Figure 2:**
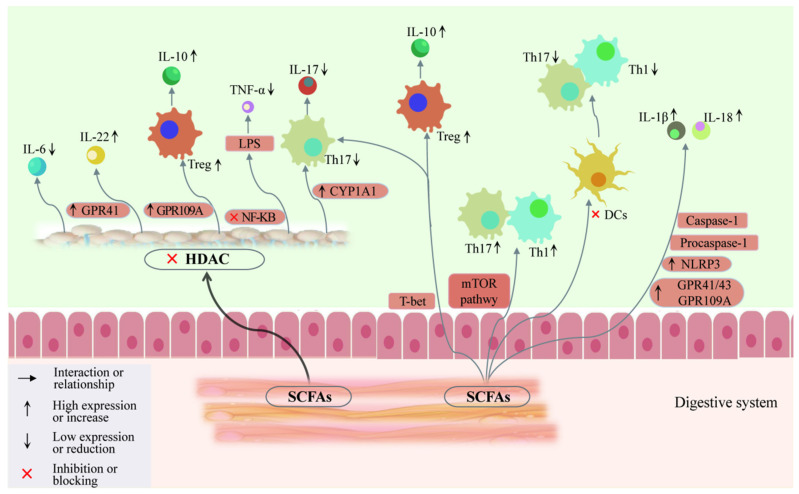
Effect of SCFAs on T cell subset differentiation and immune cytokine secretion.

Gas chromatography–mass spectrometry studies have shown that patients with AITDs have significantly lower levels of propionate and butyrate, with elevated isovalerate levels. Propionate negatively correlates with thyroid hormones FT3, FT4, and TRAb levels, and positively correlates with TSH levels. SCFAs interact with GPCRs (GPR41, GPR43, and GPR109A) on leukocytes and intestinal epithelial cells, inhibiting AITDs (Figure 2), increasing GPR41, GPR109A [85], and aryl hydrocarbon receptor (AhR) expression, and promoting the differentiation of CD4^+^ T cells. They also upregulate IL-22 and IL-10 expression, suppress AITDs and NF-κB inflammatory pathways, reduce lipopolysaccharide stimulation, and modulate TNF-α expression.

**Figure 3:**
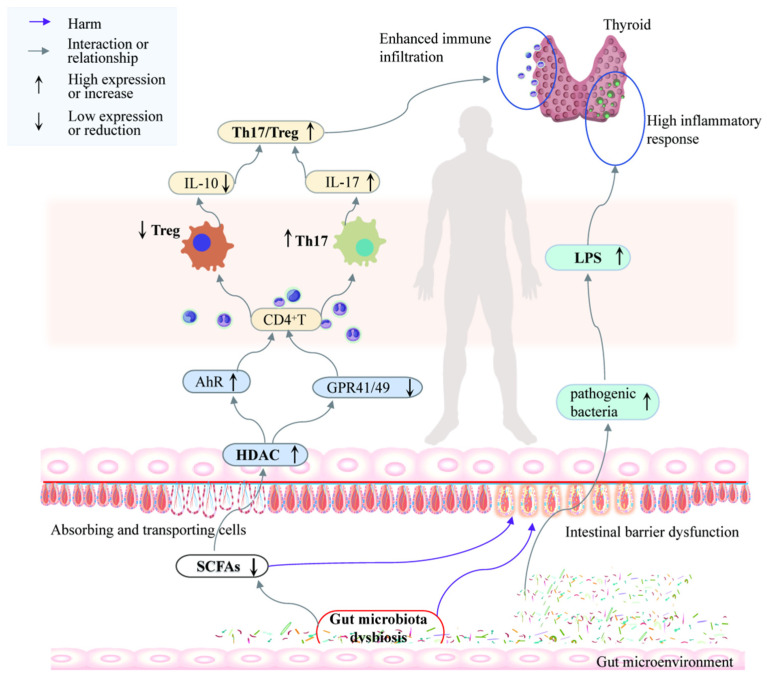
The regulatory role of the gut microbiota in AITDs. In AITD patients, there is an upregulation of IL-17 expression and a dysregulation of the intestinal microbiome. The imbalance in the intestinal flora disrupts intestinal innate immunity via SCFAs, interferes with T cell subset differentiation, decreases Treg cell numbers, and downregulates IL-10 expression. These alterations collectively contribute to the development and progression of AITDs.

Furthermore, SCFAs play a significant role in immune regulation by influencing the expression of the FoxP3 gene through increased histone acetylation, which aids in the differentiation of extrathymic Treg cells [86]. They also stimulate cells to produce IL-10 while reducing the production of pro-inflammatory chemokines such as CXCL11 and CCL3 [87]. Specifically, butyrate blocks pro-inflammatory Th1 cells in the thyroid via the PPAR-c signaling pathway [88]. Administering butyrate via gavage in mice effectively reduces NF-κB activation in the colonic mucosa, thereby modulating AITD-related inflammation [89]. Following the onset of an AITD, inflammation in the thyroid gland leads to an increase in Th17 cells among the immune cells, while imbalanced thyroid hormone levels decrease the diversity and abundance of the gut microbiota, reducing the production of SCFAs. This reduction impairs gut function and triggers disorders in both adaptive and innate immune responses within the intestinal immune system. Lower SCFA levels weaken the inhibition of HDACs and the activation of GPCRs, failing to activate GPR43 and subsequently failing to promote NLRP3 inflammasome activation and neutrophil attraction to inflammation sites. Simultaneously, increased HDAC3 and HDAC6 levels inhibit NF-κB, PPARγ, and AHR activation, leading to reduced innate lymphoid cells (ILCs) and IL-22 production. Additionally, low SCFA levels hinder the promotion of intestinal Treg cell differentiation and the inhibition of the IL-6–STAT3–IL-17 pathway, resulting in decreased IL-10 secretion and increased IL-17 production, thus disrupting the Th17/Treg balance. In summary, the reduction in gut microbial abundance and diversity, influenced by the SCFA levels, shifts the Th17/Treg ratio towards Th17, increasing the inflammatory factor expression and further contributing to AITDs (Figure 3).

## 6. Involvement of Gut Microbiota and Metabolites in AITDs through Thyroid Hormones

### 6.1. Gut Microbiota Is Significantly Associated with Thyroid Hormones

Dietary interventions, prebiotics, probiotics, microbial treatments, and fecal microbiota transplantation have shown that variations in the abundance of the gut microbiota, such as *Prevotella*, *Bacteroides*, and *Bifidobacterium*, can influence AITD-related thyroid hormone expression. Studies on gut microbiota sequencing in AITD patients have shown significant connections between microbial levels and markers such as TPOAbs, TRAbs, TGAbs, and TSH [90]. Genera, such as *Ruminococcus* and *Clostridium*, were positively correlated with TSH, whereas *Streptococcus* and *Veillonella* were negatively correlated with TSH [91]. Additionally, *Limibacter* [36] and *Synergistetes* [17] are negatively correlated with TSAbs. *Lactobacillus*, *Ruminococcus*, *Bifidobacterium*, and *Veillonella* are positively correlated with TRAbs [43]. *Prevotella 9*, *Actinomyces odontolyticus*, *Selenomonadales*, *Coriobacteriia*, *Collinsella*, and *Negativicutes* are positively correlated with TPOAbs [9]. *Romboutsia*, *Lachnoclostridium*, *Monoglobus*, *Haemophilus*, and *Streptococcus* are related to FT3 [49]. *Bifidobacterium*, *Bacteroides* are positively correlated with FT4 [35]. *Roseburia*, *Oscillibacter*, *Odoribacter*, *Flavonifractor*, *Escherichia-Shigella*, and *Eubacterium ventriosum group* are negatively correlated with TRAbs [35]. *Dorea*, *Faecalibacterium*, and *Coprococcus* are negatively correlated with TPOAbs [17]. *Clostridia UCG 014*, *Faecalibacterium*, *Lachnospiraceae NK4A136 group*, *Eubacterium ruminantium group*, and *Lachnospiraceae UCG 001* are negatively correlated with FT4 [49].

### 6.2. Involvement in AITDs through Regulation of Thyroid Hormones

The gut microbiota significantly influences the absorption of essential nutrients from various food sources, including vegetables, fruits, proteins, dairy products, fatty acids, and carbohydrates. These nutrients facilitate the synthesis of vital trace elements such as iodine, selenium, zinc, and iron [92,93]. Iodine, essential for synthesizing thyroid hormones, is primarily absorbed through the gastrointestinal tract [94]. It is primarily concentrated in the thyroid gland, and is crucial for maintaining thyroid health [95]. Iodine deficiencies are linked to poor development in children, while excess iodine can lead to increased Th17 cell counts in thyroid immune infiltrates [96], cause oxidative stress, damage thyroid tissue, and enhance the immunogenicity of thyroglobulin. Thus, the thyroid gland relies on trace elements to maintain its normal function by synthesizing and metabolizing thyroid hormones [97]. Tg, a glycosylated protein, is secreted by thyroid cells, accumulating in the thyroid follicular lumen, and is metabolized to release triiodothyronine (T3) and T4 hormones [7]. Animal studies have shown that excess iodine can reduce circulating thyroid hormones and TSH levels in mice while increasing FT3 and FT4 levels [63]. Iodine supplementation has also been found to reduce the populations of *Prausnitzii*, *fecal bacilli*, *Lactobacillus*, and *Bifidobacterium* [98]. SCFAs, along with the sodium/iodide cotransporter, can increase the iodine uptake of thyroid follicular cells [99].

The thyroid gland contains the highest concentration of selenium in the human body, yet patients with HT often exhibit low selenium levels [100]. Selenium-dependent iodothyronine deiodinase, which is crucial for thyroid cell metabolism, catalyzes the conversion of T4 to T3. Selenium enhances the activity of CD4^+^ CD25^+^ FoxP3^+^ Treg cells, reduces cytokine secretion, and prevents follicular cell apoptosis [101]. Selenium deficiency impairs thyroid hormone synthesis and plays a vital role in cell permeability, redox balance, and the regulation of inflammatory cell infiltration [102]. Selenium supplementation has been shown to reduce anti-thyroid antibody levels in patients with AITDs. In mice, selenium deficiency has been shown to trigger an intestinal immune response and barrier dysfunction, resulting in thyroid dysfunction [103]. Intestinal microbes such as *Bacteroides* convert intracellular selenite into selenocysteine and selenomethionine, aiding the body in absorbing selenium in organic forms [104]. Patients with AITDs often have lower levels of iron, folic acid, zinc, and vitamins. Research indicates that patients with positive thyroid autoantibodies have significantly lower levels of hemoglobin, ferritin, and iron compared to healthy individuals, with a negative correlation between TPOAbs and serum ferritin and iron levels [105]. TPO, a heme enzyme necessary for thyroid hormone production, becomes active when it binds with heme. Iron acts as a coenzyme for TPO, which is necessary for catalyzing the iodination and coupling reactions that combine iodine with tyrosine to form T3 and T4 [106]. Iron imbalances affect the differentiation of Th1, Th2, and Th17 cells [107]. In cases of hypoferremia, the expression of Th17 cells in the spleen and lymph nodes of mice increases. Supplementing with hepcidin can reverse the inflammation response induced by LPS. Additionally, elevated iron levels promote the production of TNF-α and exacerbate oxidative stress, leading to thyroid tissue damage and an enhanced immune response. Both animal and human studies show that zinc deficiency affects thyroid metabolism, with notably lower zinc levels in patients with hypothyroidism [108]. Zinc plays a role in the synthesis of TSH via the hypothalamus–pituitary–thyroid axis, influencing T3 and T4 levels [109]. Zinc deficiency also reduces the production of SCFAs. Dietary zinc supplementation has several benefits, including enhancing immune function, improving thyroid function by lowering TSH levels, and inhibiting NF-κB activation in immune cells and inflammatory factor production [110].

**Figure 4:**
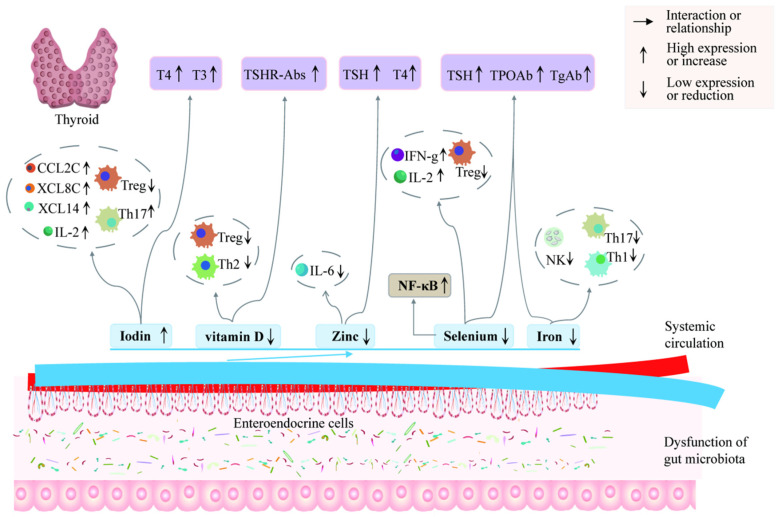
The effects of essential trace elements on thyroid hormones. Iodine is crucial for the production of the thyroid hormones T3 and T4. Selenium is vital for maintaining the activity and antioxidant properties of these hormones. Iron acts as a coenzyme for TPO, which is necessary for catalyzing the iodination and coupling reactions that combine iodine with tyrosine to form T3 and T4. Zinc also serves as a cofactor for TPO, facilitating the iodination process. Additionally, vitamin D plays a role in thyroid health by regulating the immune system and enhancing calcium absorption.

It is worth noting that thyroid follicular cells contain vitamin D receptors. Patients with AITDs tend to have more severe vitamin D deficiencies following thyroid inflammation compared to the general population [111]. Vitamin D promotes immune tolerance and regulates CD4^+^ T cell differentiation. It primarily acts on antigen-presenting cells and T cells, inhibiting the proliferation of antigen-specific T cells and cytokine production [112,113]. Additionally, vitamin D boosts Th2 and Treg cell activity, while inhibiting Th1 and Th17 cell activity (Figure 4). Thyroid hormones, as essential markers of normal thyroid function, are crucial for diagnosing GD and HT. Patients with AITDs often exhibit altered storage of these trace elements, resulting in deficiencies in selenium, zinc, and vitamin D, and an excess of iodine. Therefore, dysbiosis of the gut microbiota leads to disorders in the synthesis and secretion of thyroid hormones by regulating the levels of metabolites such as iodine, selenium, iron, zinc, and vitamins, thereby exacerbating the severity of AITDs.

## 7. Summary and Prospects

AITDs occur when the immune system mistakenly attacks the thyroid gland, leading to dysfunction such as GD or HT. This dysfunction is often marked by the presence of specific antibodies against thyroid antigens in the bloodstream, including TRAbs, TPOAbs, and TGAbs. Abnormal levels of these antibodies can further disrupt thyroid function, complicating the management of AITDs. The current study highlights the comprehensive role of the gut microbiota in AITDs. The intestinal barrier, primarily composed of the intestinal epithelium, the overlying mucus layer, and the gut-associated lymphoid tissue, serves as a defense against pathogenic invasion through microbial, mechanical, chemical, and immune barriers. Toll-like receptors (TLRs) and NLRs recognize pathogen-associated molecular patterns (PAMPs) from the microbiota, including pathogenic bacteria. This recognition can trigger inflammation and activate lymphocytes. This process can lead to a complex interaction between the increased intestinal permeability and immune activation. Increased permeability may enable antigen penetration and initiate immune responses, while immune activation can further increase intestinal permeability. These changes are often reflected in the altered levels of markers such as LPS, D-lactate, and I-FABP. Previous literature suggests that patients with AITDs exhibit reduced gut microbial diversity and insufficient absorption of trace elements. However, the effects of different types, doses, and methods of trace element absorption, as well as the combined use of multiple trace elements, have not been comprehensively evaluated. Future research should explore the role of gut microbiota in the absorption of essential trace elements for thyroid function.

Despite variations in the gut microbiota composition among AITDs patients, detailed reports on its characteristics at different stages of disease progression are scarce. Existing studies often rely on fecal samples for gut microbiota analyses without considering individual differences such as genetics, environment, diet, and trace element absorption and utilization. Different treatment methods, such as probiotics, prebiotics, dietary modifications, antibiotics, trace elements, etc., may have varying effects on individuals, and the long-term consequences are not yet fully understood. Many recent studies have utilized small sample sizes and lacked multi-center, large-scale, long-term study designs, resulting in a limited understanding of the interactions and pathogenic mechanisms between the gut microbiota and AITDs. Therefore, more mechanistic studies are needed to uncover the biological relationships between the microbiota and AITDs. Data should be collected from patients with AITDs and the control groups via multi-center, large-sample clinical cohorts. Additionally, clinical translational research is needed to verify the feasibility and effectiveness of targeting the gut microbiota as a treatment for AITDs. This review strengthens the argument for the interdependency between the gut microbiota and the thyroid gland, supporting the existence of a gut–thyroid axis. Future research might focus on how the modulation of gut microbiota could improve thyroid health or serve as an adjunct strategy to treat AITDs, providing more precise treatment strategies for AITDs.

## Figures and Tables

**Table 1:** Gut microbiota characteristics of GD.

Phyla Level	Genus/Species Level		References
	Augmented:	Reduced:	
Firmicutes		*Anaerostipes*, *Faecalibacterium*	[3]
	*Clostridiaceae 02d06*, *Clostridium estertheticum*		[47]
	*Megamonas*, *Veillonella*	*Butyricimonas*, *Anaerostipes*	[37]
	*Ruminococcus*	*E.ventriosum*, *Flavonifractor*, *Romboutsia*, *Oscillospiaceae UCG 002*	[38]
	*Enterococcus*	*Roseburia*, *Dialister*	[28]
	*Anaerofilum*, *Intestinimonas*, *Peptococcus*, *Ruminococcaceae UCG 005*		[43]
		*Phascolarctobacterium*	[44]
	*Pediococcus*, *Streptococcus parasanguinis*, *Streptococcus salivarius,* *Veillonella parvula*	*Faecalibacterium*, *prausnitzii*, *Butyricimonas faecalis*	[33]
	*Ruminococcus* sp., *Dorea* sp., *Eubacterium ventriosum*		[30]
Bacteroidota	*Bacteroides*, *Prevotella 9*		[9]
	*Prevotella*		[12]
		*Odoribacter*, *Rikenellaceae*	[28]
		*Parabacteroides*	[34]
Acfinobacteria	*Actinobacillus*		[3]
		*Rothia mucilaginosa*	[47]
	*Eggerthella lenta*, *Collinsella*	*Bifdobacterium*, *Corynebacterium*	[33]

**Table 2:** Gut microbiota characteristics of HT.

Phyla Level	Genus/Species Level		References
	Augmented:	Reduced:	
Firmicutes	*Ruminococcus*, *Roseburia*, *Dorea*, *[Eubacterium] hallii group*	*Lachnoclostridium*, *Fecalibacterium*	[52]
	*Megamonas*, *Clostridia*, *Holdemania*, *oscillospirales*, *Lachnospiraceae NC2004 group*	*Faecalibacterium*	[49]
	*Enterococcus*		[41]
	*Ruminococcus 2*		[31]
	*Lachnospiraceae incertae sedis*, *Subdoligranulum*, *Subdoligranulum*		[37]
	*Granulicatella*		[54]
Bacteroidota	*Flavobacteriaceae*		[53]
	*Escherichia Shigella*, *Parasutterella Bacteroides*, *Blautia*	*Prevotella 9*	[29]
	*Paraprevotella*		[32]
Proteobacteria	*Ralstonia*, *Acetitomaculum*		[53]
	*Alistipes*		[41]
	*Veillonella*	*Neisseria*, *Rheinheimera*	[32]
	*Haemophilus*		[52]

## Data Availability

No data was used for the research described in this article.

## References

[1] Jeong C., Baek H., Bae J., Hwang N., Ha J., Cho Y.S., Lim D.J. (2024). Gut microbiome in the Graves’ disease: Comparison before and after anti-thyroid drug treatment. PLoS ONE.

[2] Grimm D. (2021). Cell and Molecular Biology of Thyroid Disorders 2.0. Int. J. Mol. Sci..

[3] Jiang W., Yu X., Kosik R.O., Song Y., Qiao T., Tong J., Liu S., Fan S., Luo Q., Chai L. (2021). Gut Microbiota May Play a Significant Role in the Pathogenesis of Graves’ Disease. Thyroid.

[4] Xi Z., Yang T., Huang T., Zhou J., Yang P. (2023). Identification and Preliminary Clinical Validation of Key Extracellular Proteins as the Potential Biomarkers in Hashimoto’s Thyroiditis by Comprehensive Analysis. Biomedicines.

[5] Hönes G.S., Sivakumar R.G., Hoppe C., König J., Führer D., Moeller L.C. (2022). Cell-Specific Transport and Thyroid Hormone Receptor Isoform Selectivity Account for Hepatocyte-Targeted Thyromimetic Action of MGL-3196. Int. J. Mol. Sci..

[6] Beduleva L., Sidorov A., Fomina K., Terentiev A., Menshikov I., Shklyaeva N., Ivanov P., Varaksin V. (2024). Experimental rat models for Hashimoto’s thyroiditis. J. Endocrinol. Investig..

[7] Zheng H., Xu J., Chu Y., Jiang W., Yao W., Mo S., Song X., Zhou J. (2022). A Global Regulatory Network for Dysregulated Gene Expression and Abnormal Metabolic Signaling in Immune Cells in the Microenvironment of Graves’ Disease and Hashimoto’s Thyroiditis. Front. Immunol..

[8] Stensland Z.C., Coleman B.M., Rihanek M., Baxter R.M., Gottlieb P.A., Hsieh E.W.Y., Sarapura V.D., Simmons K.M., Cambier J.C., Smith M.J. (2022). Peripheral immunophenotyping of AITD subjects reveals alterations in immune cells in pediatric vs. adult-onset AITD. iScience.

[9] Chang S.-C., Lin S.-F., Chen S.-T., Chang P.-Y., Yeh Y.-M., Lo F.-S., Lu J.-J. (2021). Alterations of Gut Microbiota in Patients with Graves’ Disease. Front. Cell. Infect. Microbiol..

[10] Ni Y., Hu Y., Lou X., Rong N., Liu F., Yang C., Zheng A., Yang S., Bao J., Fu Z. (2022). Spermidine Ameliorates Nonalcoholic Steatohepatitis through Thyroid Hormone-Responsive Protein Signaling and the Gut Microbiota-Mediated Metabolism of Bile Acids. J. Agric. Food Chem..

[11] Alkader D.A.A., Asadi N., Solangi U., Singh R., Rasuli S.F., Farooq M.J., Raheela F.N.U., Waseem R., Gilani S.M., Abbas K. (2023). Exploring the role of gut microbiota in autoimmune thyroid disorders: A systematic review and meta-analysis. Front. Endocrinol..

[12] El-Zawawy H.T., Ahmed S.M., El-Attar E.A., Ahmed A.A., Roshdy Y.S., Header D.A. (2021). Study of gut microbiome in Egyptian patients with autoimmune thyroid diseases. Int. J. Clin. Pract..

[13] Miro C., Nappi A., Sagliocchi S., Di Cicco E., Murolo M., Torabinejad S., Acampora L., Pastore A., Luciano P., La Civita E. (2023). Thyroid Hormone Regulates the Lipid Content of Muscle Fibers, Thus Affecting Physical Exercise Performance. Int. J. Mol. Sci..

[14] Fotakis C., Moros G., Kontogeorgou A., Iacovidou N., Boutsikou T., Zoumpoulakis P. (2022). Uncontrolled Thyroid during Pregnancy Alters the Circulative and Exerted Metabolome. Int. J. Mol. Sci..

[15] Nguyen M.T., Ly D.D., Nguyen N.T., Qi X.F., Yi H.S., Shong M., Cha S.K., Park S., Park K.S. (2021). Thyroid Hormone Induces Ca(2+)-Mediated Mitochondrial Activation in Brown Adipocytes. Int. J. Mol. Sci..

[16] Han Z., Cen C., Ou Q., Pan Y., Zhang J., Huo D., Chen K. (2021). The Potential Prebiotic Berberine Combined with Methimazole Improved the Therapeutic Effect of Graves’ Disease Patients Through Regulating the Intestinal Microbiome. Front. Immunol..

[17] Cornejo-Pareja I., Ruiz-Limón P., Gómez-Pérez A.M., Molina-Vega M., Moreno-Indias I., Tinahones F.J. (2020). Differential Microbial Pattern Description in Subjects with Autoimmune-Based Thyroid Diseases: A Pilot Study. J. Pers. Med..

[18] Wang B., Xu Y., Zhang M., Zhang J., Hou X., Li J., Cai Y., Sun Z., Ban Y., Wang W. (2020). Oral and intestinal microbial features in pregnant women with hypothyroidism and their correlations with pregnancy outcomes. Am. J. Physiol. Endocrinol. Metab..

[19] Achilla C., Chorti A., Papavramidis T., Angelis L., Chatzikyriakidou A. (2024). Genetic and Epigenetic Association of FOXP3 with Papillary Thyroid Cancer Predisposition. Int. J. Mol. Sci..

[20] Guo C., Liu Q., Zong D., Zhang W., Zuo Z., Yu Q., Sha Q., Zhu L., Gao X., Fang J. (2022). Single-cell transcriptome profiling and chromatin accessibility reveal an exhausted regulatory CD4+ T cell subset in systemic lupus erythematosus. Cell Rep..

[21] Chen Z., Liu Y., Hu S., Zhang M., Shi B., Wang Y. (2021). Decreased Treg Cell and TCR Expansion Are Involved in Long-Lasting Graves’ Disease. Front. Endocrinol..

[22] Chen J., Wang W., Guo Z., Huang S., Lei H., Zang P., Lu B., Shao J., Gu P. (2021). Associations between gut microbiota and thyroidal function status in Chinese patients with Graves’ disease. J. Endocrinol. Investig..

[23] Gong B., Wang C., Meng F., Wang H., Song B., Yang Y., Shan Z. (2021). Association between Gut Microbiota and Autoimmune Thyroid Disease: A Systematic Review and Meta-Analysis. Front. Endocrinol..

[24] Huang Y.H., Chang L.C., Chang Y.C., Chung W.H., Yang S.F., Su S.C. (2023). Compositional Alteration of Gut Microbiota in Psoriasis Treated with IL-23 and IL-17 Inhibitors. Int. J. Mol. Sci..

[25] Lin B., Zhao F., Liu Y., Wu X., Feng J., Jin X., Yan W., Guo X., Shi S., Li Z. (2022). Randomized Clinical Trial: Probiotics Alleviated Oral-Gut Microbiota Dysbiosis and Thyroid Hormone Withdrawal-Related Complications in Thyroid Cancer Patients Before Radioiodine Therapy Following Thyroidectomy. Front. Endocrinol..

[26] Bossowski A., Moniuszko M., Idźkowska E., Grubczak K., Singh P., Bossowska A., Diana T., Kahaly G.J. (2016). Decreased proportions of CD4 + IL17+/CD4 + CD25 + CD127- and CD4 + IL17+/CD4 + CD25 + CD127 - FoxP3+ T cells in children with autoimmune thyroid diseases. Autoimmunity.

[27] Cayres L.C.F., de Salis L.V.V., Rodrigues G.S.P., Lengert A.V.H., Biondi A.P.C., Sargentini L.D.B., Brisotti J.L., Gomes E., de Oliveira G.L.V. (2021). Detection of Alterations in the Gut Microbiota and Intestinal Permeability in Patients with Hashimoto Thyroiditis. Front. Immunol..

[28] Fenneman A.C., Rampanelli E., van der Spek A.H., Fliers E., Nieuwdorp M. (2023). Protocol for a double-blinded randomised controlled trial to assess the effect of faecal microbiota transplantations on thyroid reserve in patients with subclinical autoimmune hypothyroidism in the Netherlands: The IMITHOT trial. BMJ Open.

[29] Erdem M.G., Unlu O., Ates F., Karis D., Demirci M. (2023). Oral Microbiota Signatures in the Pathogenesis of Euthyroid Hashimoto’s Thyroiditis. Biomedicines.

[30] Su X., Zhao Y., Li Y., Ma S., Wang Z. (2020). Gut dysbiosis is associated with primary hypothyroidism with interaction on gut-thyroid axis. Clin. Sci..

[31] Yao Z., Zhao M., Gong Y., Chen W., Wang Q., Fu Y., Guo T., Zhao J., Gao L., Bo T. (2020). Relation of Gut Microbes and L-Thyroxine Through Altered Thyroxine Metabolism in Subclinical Hypothyroidism Subjects. Front. Cell Infect. Microbiol..

[32] Yu X., Jiang W., Kosik R.O., Song Y., Luo Q., Qiao T., Tong J., Liu S., Deng C., Qin S. (2022). Gut microbiota changes and its potential relations with thyroid carcinoma. J. Adv. Res..

[33] Demir E., Önal B., Özkan H., Utku İ.K., Şahtiyancı B., Kumbasar A., Yenmiş G., Demir B. (2022). The relationship between elevated plasma zonulin levels and Hashimoto’s thyroiditis. Turk. J. Med. Sci..

[34] Liu X., Yuan J., Liu S., Tang M., Meng X., Wang X., Li Y., Chai Y., Kou C., Yang Q. (2023). Investigating causal associations among gut microbiota, metabolites and autoimmune hypothyroidism: A univariable and multivariable Mendelian randomization study. Front. Immunol..

[35] Deng Y., Wang J., Xie G., Zou G., Li S., Zhang J., Cai W., Xu J. (2023). Correlation between gut microbiota and the development of Graves’ disease: A prospective study. iScience.

[36] Moshkelgosha S., Verhasselt H.L., Masetti G., Covelli D., Biscarini F., Horstmann M., Daser A., Westendorf A.M., Jesenek C., Philipp S. (2021). Modulating gut microbiota in a mouse model of Graves’ orbitopathy and its impact on induced disease. Microbiome.

[37] Liu S., An Y., Cao B., Sun R., Ke J., Zhao D. (2020). The Composition of Gut Microbiota in Patients Bearing Hashimoto’s Thyroiditis with Euthyroidism and Hypothyroidism. Int. J. Endocrinol..

[38] Zhang Q., Tong B., Xie Z., Li Y., Li Y., Wang L., Luo B., Qi X. (2023). Changes in the gut microbiota of patients with Graves’ orbitopathy according to severity grade. Clin. Exp. Ophthalmol..

[39] Yan H.X., An W.C., Chen F., An B., Pan Y., Jin J., Xia X.P., Cui Z.J., Jiang L., Zhou S.J. (2020). Intestinal microbiota changes in Graves’ disease: A prospective clinical study. Biosci. Rep..

[40] Biscarini F., Masetti G., Muller I., Verhasselt H.L., Covelli D., Colucci G., Zhang L., Draman M.S., Okosieme O., Taylor P. (2023). Gut Microbiome Associated with Graves Disease and Graves Orbitopathy: The INDIGO Multicenter European Study. J. Clin. Endocrinol. Metab..

[41] Cao J., Wang N., Luo Y., Ma C., Chen Z., Chenzhao C., Zhang F., Qi X., Xiong W. (2023). A cause–effect relationship between Graves’ disease and the gut microbiome contributes to the thyroid–gut axis: A bidirectional two-sample Mendelian randomization study. Front. Immunol..

[42] Zhao H., Yuan L., Zhu D., Sun B., Du J., Wang J. (2022). Alterations and Mechanism of Gut Microbiota in Graves’ Disease and Hashimoto’s Thyroiditis. Pol. J. Microbiol..

[43] Yang M., Zheng X., Wu Y., Zhang R., Yang Q., Yu Z., Liu J., Zha B., Gong Q., Yang B. (2022). Preliminary Observation of the Changes in the Intestinal Flora of Patients with Graves’ Disease Before and After Methimazole Treatment. Front. Cell. Infect. Microbiol..

[44] Zhu Q., Hou Q., Huang S., Ou Q., Huo D., Vázquez-Baeza Y., Cen C., Cantu V., Estaki M., Chang H. (2021). Compositional and genetic alterations in Graves’ disease gut microbiome reveal specific diagnostic biomarkers. ISME J..

[45] Zheng D., Liao H., Chen S., Liu X., Mao C., Zhang C., Meng M., Wang Z., Wang Y., Jiang Q. (2021). Elevated Levels of Circulating Biomarkers Related to Leaky Gut Syndrome and Bacterial Translocation Are Associated with Graves’ Disease. Front. Endocrinol..

[46] Li A., Li T., Gao X., Yan H., Chen J., Huang M., Wang L., Yin D., Li H., Ma R. (2021). Gut Microbiome Alterations in Patients with Thyroid Nodules. Front. Cell. Infect. Microbiol..

[47] Huo D., Cen C., Chang H., Ou Q., Jiang S., Pan Y., Chen K., Zhang J. (2021). Probiotic Bifidobacterium longum supplied with methimazole improved the thyroid function of Graves’ disease patients through the gut-thyroid axis. Commun Biol.

[48] Cai Y., Xu Y., Ban Y., Li J., Sun Z., Zhang M., Wang B., Hou X., Hao Y., Ouyang Q. (2022). Plasma Lipid Profile and Intestinal Microflora in Pregnancy Women with Hypothyroidism and Their Correlation with Pregnancy Outcomes. Front. Endocrinol..

[49] Liu J., Qin X., Lin B., Cui J., Liao J., Zhang F., Lin Q. (2022). Analysis of gut microbiota diversity in Hashimoto’s thyroiditis patients. BMC Microbiol..

[50] Dong T., Zhao F., Yuan K., Zhu X., Wang N., Xia F., Lu Y., Huang Z. (2021). Association between Serum Thyroid-Stimulating Hormone Levels and Salivary Microbiome Shifts. Front. Cell. Infect. Microbiol..

[51] DeClercq V., Nearing J.T., Langille M.G.I. (2021). Investigation of the impact of commonly used medications on the oral microbiome of individuals living without major chronic conditions. PLoS ONE.

[52] Zhao F., Feng J., Li J., Zhao L., Liu Y., Chen H., Jin Y., Zhu B., Wei Y. (2018). Alterations of the Gut Microbiota in Hashimoto’s Thyroiditis Patients. Thyroid.

[53] Zhou Y., Xu H., Xu J., Guo X., Zhao H., Chen Y., Zhou Y., Nie Y.F. (2021). prausnitzii and its supernatant increase SCFAs-producing bacteria to restore gut dysbiosis in TNBS-induced colitis. AMB Express.

[54] Balakrishnan B., Luckey D., Bodhke R., Chen J., Marietta E., Jeraldo P., Murray J., Taneja V. (2021). Prevotella histicola Protects From Arthritis by Expansion of Allobaculum and Augmenting Butyrate Production in Humanized Mice. Front. Immunol..

[55] Sun M., Wu W., Chen L., Yang W., Huang X., Ma C., Chen F., Xiao Y., Zhao Y., Ma C. (2018). Microbiota-derived short-chain fatty acids promote Th1 cell IL-10 production to maintain intestinal homeostasis. Nat. Commun..

[56] Haghikia A., Jörg S., Duscha A., Berg J., Manzel A., Waschbisch A., Hammer A., Lee D.H., May C., Wilck N. (2015). Dietary Fatty Acids Directly Impact Central Nervous System Autoimmunity via the Small Intestine. Immunity.

[57] Sanchez H.N., Moroney J.B., Gan H., Shen T., Im J.L., Li T., Taylor J.R., Zan H., Casali P. (2020). B cell-intrinsic epigenetic modulation of antibody responses by dietary fiber-derived short-chain fatty acids. Nat. Commun..

[58] Luo M., Chen Y., Pan X., Chen H., Fan L., Wen Y. (2023). *E. coli* Nissle 1917 ameliorates mitochondrial injury of granulosa cells in polycystic ovary syndrome through promoting gut immune factor IL-22 via gut microbiota and microbial metabolism. Front. Immunol..

[59] Lou K., Liu S., Zhang F., Sun W., Su X., Bi W., Yin Q., Qiu Y., Zhang Z., Jing M. (2023). The effect of hyperthyroidism on cognitive function, neuroinflammation, and necroptosis in APP/PS1 mice. J. Transl. Med..

[60] Zhang M., Jiang W., Lu G., Wang R., Lv Z., Li D. (2022). Insight Into Mouse Models of Hyperthyroidism. Front. Endocrinol..

[61] Zekri Y., Guyot R., Flamant F. (2022). An Atlas of Thyroid Hormone Receptors’ Target Genes in Mouse Tissues. Int. J. Mol. Sci..

[62] Li Z., Wenhart C., Reimann A., Goebel S., Cho Y.L., Muench G. (2024). Mapping Thyroid Changes in Size and Position During Enlargement in Adult Mice with Hyperthyroidism. Endocrinology.

[63] Shen H., Han J., Li Y., Lu C., Zhou J., Li Y., Su X. (2019). Different host-specific responses in thyroid function and gut microbiota modulation between diet-induced obese and normal mice given the same dose of iodine. Appl. Microbiol. Biotechnol..

[64] Yuan Q., Zhao Y., Zhu X., Liu X. (2016). Low regulatory T cell and high IL-17 mRNA expression in a mouse Graves’ disease model. J. Endocrinol. Investig..

[65] Liu X., Jiang W., Lu G., Qiao T., Gao D., Zhang M., Cai H., Chai L., Yi W., Lv Z. (2023). The Potential Role of Pyrroloquinoline Quinone to Regulate Thyroid Function and Gut Microbiota Composition of Graves’ Disease in Mice. Pol. J. Microbiol..

[66] Su X., Yin X., Liu Y., Yan X., Zhang S., Wang X., Lin Z., Zhou X., Gao J., Wang Z. (2020). Gut Dysbiosis Contributes to the Imbalance of Treg and Th17 Cells in Graves’ Disease Patients by Propionic Acid. J. Clin. Endocrinol. Metab..

[67] Ferreira R.L.U., Sena-Evangelista K.C.M., de Azevedo E.P., Pinheiro F.I., Cobucci R.N., Pedrosa L.F.C. (2021). Selenium in Human Health and Gut Microflora: Bioavailability of Selenocompounds and Relationship with Diseases. Front. Nutr..

[68] Misharin A., Hewison M., Chen C.R., Lagishetty V., Aliesky H.A., Mizutori Y., Rapoport B., McLachlan S.M. (2009). Vitamin D deficiency modulates Graves’ hyperthyroidism induced in BALB/c mice by thyrotropin receptor immunization. Endocrinology.

[69] González-Ramos S., Paz-García M., Fernández-García V., Portune K.J., Acosta-Medina E.F., Sanz Y., Castrillo A., Martín-Sanz P., Obregon M.J., Boscá L. (2020). NOD1 deficiency promotes an imbalance of thyroid hormones and microbiota homeostasis in mice fed high fat diet. Sci. Rep..

[70] Li J., Xu Y., Cai Y., Zhang M., Sun Z., Ban Y., Zhai S., Hao Y., Ouyang Q., Wu B. (2022). Association of Differential Metabolites with Small Intestinal Microflora and Maternal Outcomes in Subclinical Hypothyroidism During Pregnancy. Front. Cell. Infect. Microbiol..

[71] Song X., Zhang H., Zhang Y., Goh B., Bao B., Mello S.S., Sun X., Zheng W., Gazzaniga F.S., Wu M. (2023). Gut microbial fatty acid isomerization modulates intraepithelial T cells. Nature.

[72] Li X., Hu S., Yin J., Peng X., King L., Li L., Xu Z., Zhou L., Peng Z., Ze X. (2023). Effect of synbiotic supplementation on immune parameters and gut microbiota in healthy adults: A double-blind randomized controlled trial. Gut Microbes.

[73] Wu B., Xu Y., Ban Y., Zhang M., Sun Z., Cai Y., Li J., Hao Y., Ouyang Q., Hu L. (2023). Correlation between the intestinal microflora and peripheral blood Th1/Th2 balance in hypothyroidism during the first half of pregnancy. Front. Cell. Infect. Microbiol..

[74] Yang M., Li F., Zhang R., Wu Y., Yang Q., Wang F., Yu Z., Liu J., Cha B., Gong Q. (2022). Alteration of the Intestinal Microbial Flora and the Serum IL-17 Level in Patients with Graves’ Disease Complicated with Vitamin D Deficiency. Int. Arch. Allergy Immunol..

[75] Xu B., Fu Y., Yin N., Qin W., Huang Z., Xiao W., Huang H., Mei Q., Fan J., Zeng Y. (2024). Bacteroides thetaiotaomicron and Faecalibacterium prausnitzii served as key components of fecal microbiota transplantation to alleviate colitis. Am. J. Physiol. Gastrointest. Liver Physiol..

[76] Wastyk H.C., Fragiadakis G.K., Perelman D., Dahan D., Merrill B.D., Yu F.B., Topf M., Gonzalez C.G., Van Treuren W., Han S. (2021). Gut-microbiota-targeted diets modulate human immune status. Cell.

[77] Li H.B., Xu M.L., Xu X.D., Tang Y.Y., Jiang H.L., Li L., Xia W.J., Cui N., Bai J., Dai Z.M. (2022). Faecalibacterium prausnitzii Attenuates CKD via Butyrate-Renal GPR43 Axis. Circ. Res..

[78] Robles-Vera I., de la Visitación N., Toral M., Sánchez M., Romero M., Gómez-Guzmán M., Yang T., Izquierdo-García J.L., Guerra-Hernández E., Ruiz-Cabello J. (2020). Probiotic Bifidobacterium breve prevents DOCA-salt hypertension. FASEB J. Off. Publ. Fed. Am. Soc. Exp. Biol..

[79] Sun J., Zhao F., Lin B., Feng J., Wu X., Liu Y., Zhao L., Zhu B., Wei Y. (2020). Gut Microbiota Participates in Antithyroid Drug Induced Liver Injury Through the Lipopolysaccharide Related Signaling Pathway. Front. Pharmacol..

[80] Yamamura R., Nakamura K., Kitada N., Aizawa T., Shimizu Y., Nakamura K., Ayabe T., Kimura T., Tamakoshi A. (2020). Associations of gut microbiota, dietary intake, and serum short-chain fatty acids with fecal short-chain fatty acids. Biosci. Microbiota Food Health.

[81] Kircher B., Woltemate S., Gutzki F., Schlüter D., Geffers R., Bähre H., Vital M. (2022). Predicting butyrate- and propionate-forming bacteria of gut microbiota from sequencing data. Gut Microbes.

[82] Chen J., Qin Q., Yan S., Yang Y., Yan H., Li T., Wang L., Gao X., Li A., Ding S. (2021). Gut Microbiome Alterations in Patients with Carotid Atherosclerosis. Front. Cardiovasc. Med..

[83] Liang L., Liu L., Zhou W., Yang C., Mai G., Li H., Chen Y. (2022). Gut microbiota-derived butyrate regulates gut mucus barrier repair by activating the macrophage/WNT/ERK signaling pathway. Clin. Sci..

[84] Wang G., Qin S., Chen L., Geng H., Zheng Y., Xia C., Yao J., Deng L. (2023). Butyrate dictates ferroptosis sensitivity through FFAR2-mTOR signaling. Cell Death Dis..

[85] Tian S., Lei Y., Zhao F., Che J., Wu Y., Lei P., Kang Y.E., Shan Y. (2024). Improving insulin resistance by sulforaphane via activating the Bacteroides and Lactobacillus SCFAs-GPR-GLP1 signal axis. Food Funct..

[86] Dong L., Xu Z., Huang G., Zhang R., Deng M., Huang F., Su D. (2023). Lychee Pulp-Derived Dietary Fiber-Bound Phenolic Complex Upregulates the SCFAs-GPRs-ENS Pathway and Aquaporins in Loperamide-Induced Constipated Mice by Reshaping Gut Microbiome. J. Agric. Food Chem..

[87] Phung C.D., Tran T.H., Nguyen H.T., Nguyen T.T., Jeong J.H., Ku S.K., Yong C.S., Choi H.G., Kim J.O. (2021). Nanovaccines silencing IL-10 production at priming phase for boosting immune responses to melanoma. J. Control. Release Off. J. Control. Release Soc..

[88] Antonelli A., Ferrari S.M., Frascerra S., Ruffilli I., Gelmini S., Minuto M., Pupilli C., Miccoli P., Sellari-Franceschini S., Ferrannini E. (2012). Peroxisome proliferator-activated receptor-α agonists modulate CXCL9 and CXCL11 chemokines in Graves’ ophthalmopathy fibroblasts and preadipocytes. Mol. Cell. Endocrinol..

[89] Tian X., Zeng Y., Tu Q., Jiao Y., Yao S., Chen Y., Sun L., Xia Q., Luo Y., Yuan L. (2023). Butyrate alleviates renal fibrosis in CKD by regulating NLRP3-mediated pyroptosis via the STING/NF-κB/p65 pathway. Int. Immunopharmacol..

[90] Klasson C.L., Sadhir S., Pontzer H. (2022). Daily physical activity is negatively associated with thyroid hormone levels, inflammation, and immune system markers among men and women in the NHANES dataset. PLoS ONE.

[91] Zhou L., Li X., Ahmed A., Wu D., Liu L., Qiu J., Yan Y., Jin M., Xin Y. (2014). Gut microbe analysis between hyperthyroid and healthy individuals. Curr. Microbiol..

[92] Jiang W., Lu G., Gao D., Lv Z., Li D. (2022). The relationships between the gut microbiota and its metabolites with thyroid diseases. Front. Endocrinol..

[93] Wang B., Xu Y., Hou X., Li J., Cai Y., Hao Y., Ouyang Q., Wu B., Sun Z., Zhang M. (2021). Small Intestinal Bacterial Overgrowth in Subclinical Hypothyroidism of Pregnant Women. Front. Endocrinol..

[94] Shen H., Xu J., Lu C., Han J., Zhou J., Ming T., Li Y., Su X. (2021). Effects of the Sex Factor on Mouse Iodine Intake: Interactions between the Gut Microbiota Composition and Metabolic Syndromes. ACS Omega.

[95] Laurino A., Raimondi L. (2022). Thyroid Homeostasis: An Intricate Network of Production, Transport, Metabolism and Receptors Interaction. Int. J. Mol. Sci..

[96] McDonald C.M., Brown K.H., Goh Y.E., Manger M.S., Arnold C.D., Krebs N.F., Westcott J., Long J.M., Gibson R.S., Jamwal M. (2022). Quintuply-fortified salt for the improvement of micronutrient status among women of reproductive age and preschool-aged children in Punjab, India: Protocol for a randomized, controlled, community-based trial. BMC Nutr..

[97] Henjum S., Groufh-Jacobsen S., Aakre I., Gjengedal E.L.F., Langfjord M.M., Heen E., Sele V., Andersson M. (2023). Thyroid function and urinary concentrations of iodine, selenium, and arsenic in vegans, lacto-ovo vegetarians and pescatarians. Eur. J. Nutr..

[98] Zheng L., Zhang L., Tang L., Huang D., Pan D., Guo W., He S., Huang Y., Chen Y., Xiao X. (2023). Gut microbiota is associated with response to (131)I therapy in patients with papillary thyroid carcinoma. Eur. J. Nucl. Med. Mol. Imaging.

[99] Mendoza-León M.J., Mangalam A.K., Regaldiz A., González-Madrid E., Rangel-Ramírez M.A., Álvarez-Mardonez O., Vallejos O.P., Méndez C., Bueno S.M., Melo-González F. (2023). Gut microbiota short-chain fatty acids and their impact on the host thyroid function and diseases. Front. Endocrinol..

[100] Huang H.J., Wang S.S., Jin M.M., Cheng B.W., Liu Y., Liu X.C., Yu Q.Y., Yang X.J. (2023). Genetically predicted selenium concentrations and thyroid function: A two-sample Mendelian randomization study. Clin. Endocrinol..

[101] Vaivode I., Zake T., Strele I., Upmale-Engela S., Gogins D., Gersone G., Skesters A., Dambrova M., Konrade I. (2023). Stress-Related Immune Response and Selenium Status in Autoimmune Thyroid Disease Patients. Int. J. Mol. Sci..

[102] Sun Q., Oltra E., Dijck-Brouwer D.A.J., Chillon T.S., Seemann P., Asaad S., Demircan K., Espejo-Oltra J.A., Sánchez-Fito T., Martín-Martínez E. (2023). Autoantibodies to selenoprotein P in chronic fatigue syndrome suggest selenium transport impairment and acquired resistance to thyroid hormone. Redox Biol..

[103] Lossow K., Renko K., Schwarz M., Schomburg L., Schwerdtle T., Kipp A.P. (2021). The Nutritional Supply of Iodine and Selenium Affects Thyroid Hormone Axis Related Endpoints in Mice. Nutrients.

[104] Zavros A., Andreou E., Aphamis G., Bogdanis G.C., Sakkas G.K., Roupa Z., Giannaki C.D. (2023). The Effects of Zinc and Selenium Co-Supplementation on Resting Metabolic Rate, Thyroid Function, Physical Fitness, and Functional Capacity in Overweight and Obese People under a Hypocaloric Diet: A Randomized, Double-Blind, and Placebo-Controlled Trial. Nutrients.

[105] Wang F., Zhang Y., Yuan Z., Li Y., Liu S., Zeng X., Qiu X., Ye L., Huang D. (2022). The association between iron status and thyroid hormone levels during pregnancy. J. Trace Elem. Med. Biol. Organ Soc. Miner. Trace Elem..

[106] Shimizu Y., Matsuyama M., Noguchi Y., Takada M., Kawashiri S.Y., Fukui S., Nakamichi S., Nagata Y., Maeda T., Hayashida N. (2023). Association between anti-thyroid peroxidase antibody and thyroid stimulating hormone: A cross-sectional study. Sci. Rep..

[107] McKay A.K.A., Peeling P., Pyne D.B., Tee N., Whitfield J., Sharma A.P., Heikura I.A., Burke L.M. (2022). Six Days of Low Carbohydrate, Not Energy Availability, Alters the Iron and Immune Response to Exercise in Elite Athletes. Med. Sci. Sports Exerc..

[108] Rabbani E., Golgiri F., Janani L., Moradi N., Fallah S., Abiri B., Vafa M. (2021). Randomized Study of the Effects of Zinc, Vitamin A, and Magnesium Co-supplementation on Thyroid Function, Oxidative Stress, and hs-CRP in Patients with Hypothyroidism. Biol. Trace Elem. Res..

[109] Lu L., Huang Z., Wang X., Chen J. (2023). Interaction between Dietary Selenium and Zinc Intakes on Hypothyroidism. Biol. Trace Elem. Res..

[110] Kijima K., Ono G., Kobayakawa K., Saiwai H., Hara M., Yoshizaki S., Yokota K., Saito T., Tamaru T., Iura H. (2023). Zinc deficiency impairs axonal regeneration and functional recovery after spinal cord injury by modulating macrophage polarization via NF-κB pathway. Front. Immunol..

[111] Chen Y., Zhang S., Hu L., Dong L., Liu Q., Liu Y., Cheng W., Liu D., Yang G., Li K. (2022). Vitamin D categories and postpartum thyroid function in women with hypothyroidism. Front. Nutr..

[112] Català-Moll F., Ferreté-Bonastre A.G., Godoy-Tena G., Morante-Palacios O., Ciudad L., Barberà L., Fondelli F., Martínez-Cáceres E.M., Rodríguez-Ubreva J., Li T. (2022). Vitamin D receptor, STAT3, and TET2 cooperate to establish tolerogenesis. Cell Rep..

[113] Maciejewska-Markiewicz D., Kochman J., Jakubczyk K., Bargiel P., Szlosser Z., Stachowska E., Markowska M., Bucka A., Czapla N., Petriczko J. (2023). Vitamin D Status in Patients before Thyroidectomy. Int. J. Mol. Sci..

